# Endoscopic endonasal access for the treatment of Vidian nerve schwannoma: a case report^☆^

**DOI:** 10.1016/j.bjorl.2016.04.015

**Published:** 2016-06-04

**Authors:** Bibiana Fortes, André Beer-Furlan, Leonardo Balsalobre, Eduardo Vellutini, Aldo Stamm

**Affiliations:** a Hospital Edmundo Vasconcelos, Centro de Otorrinolaringologia e Fonoaudiologia, São Paulo, SP, Brazil; b DFV Neuro, Serviço em Neurologia e Neurocirurgia, São Paulo, SP, Brazil

## Introduction

Schwannomas are benign tumors of Schwann cells of the nerve sheath and constitute 8% of intracranial primary neoplasms. The most common origin is the vestibular nerve^1^ located in the cerebellopontine angle, corresponding to 51% of all tumors of the nerve sheath. The second most frequent type of schwannoma has its origin in the trigeminal nerve and may represent 8% of all schwannomas.

Vidian nerve schwannomas are extremely rare lesions and only seven cases have been reported in the literature.^1^, ^2^, ^3^, ^4^, ^5^, ^6^

Vidian schwannomas symptoms can result from the compression of adjacent structures and may present as headaches, facial pain and paresthesia. Vidian nerve dysfunction can cause nasal dryness and decreased lacrimation.

Knowledge of this type of tumor and its clinical presentation is important in the differential diagnosis and surgical planning of skull-base tumors, more precisely those in the pterygopalatine fossa.

## Case report

**Figure 1:**
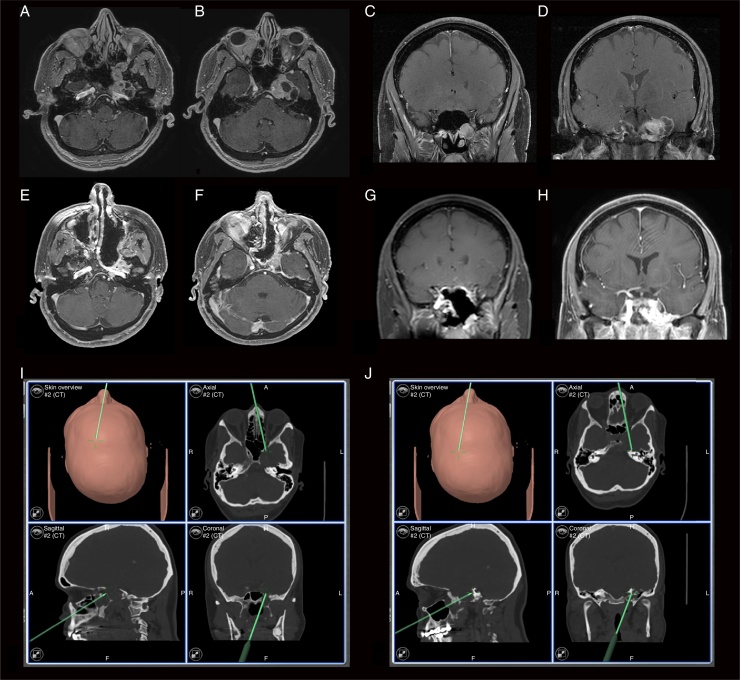
(A and B) Preoperative axial T1 MRI images with gadolinium; (C and D), preoperative coronal T1 MRI images with gadolinium; (E and F), postoperative axial T1 MRI images with gadolinium; (G and H), postoperative coronal T1 MRI images with gadolinium; (I), intraoperative neuronavigation image, showing the extension of the Vidian nerve canal (coronal); (J), intraoperative neuronavigation image showing posterior extent after lesion resection.

A previously healthy 60-year-old female patient came to our service complaining of reduced sensitivity in the left facial region for two months. On examination, the patient had hypoesthesia in the territory of the maxillary (V2) and mandibular (V3) branches of the trigeminal nerve. She had no other complaints or alterations at the physical examination. The radiological investigation carried out with cranial computed tomography and MRI showed a left-sided ventral and paramedian solid-cystic mass lesion in the skull base that exhibited heterogeneous contrast enhancement. There was intra and extracranial extension through the Vidian nerve canal, which was enlarged (Fig. 1).

We opted for the surgical endoscopic endonasal treatment aiming to reduce the expansive effect of the lesion and for diagnostic confirmation. The transpterygoid access was performed to left, making it possible to preserve the vascular pedicle and to create an ipsilateral nasoseptal flap. The tumor could be visualized in the sphenoid sinus and pterygopalatine fossa, distorting the local anatomy. It medially displaced the paraclival segment of the internal carotid artery and, laterally, the trigeminal nerve and Gasserian ganglion. After opening the tumor pseudocapsule, the lesion core was emptied using an aspirator. Once decompressed, the endoscopic microsurgical technique was used for dissection of the tumor. During the procedure, it was verified that V2 and V3 were tumor-free and the lesion was fully extradural and the dura mater of the middle fossa was intact. Subsequently, the extension of the tumor above and below the petrous portion (horizontal) of the internal carotid artery was identified and removed.

The patient required nasal packing for 48 h. On the fourth postoperative day, the patient was discharged, without complications. Ten days after the surgery she returned to the clinic and reported facial hypoesthesia improvement. Histopathological analysis confirmed the diagnosis of schwannoma. The MRI performed three months after the surgery showed complete lesion resection.

## Discussion

Vidian nerve schwannoma is an extremely rare tumor. Including this case report, there have been eight cases reported in the literature.

The Vidian nerve or nerve of the pterygoid canal consists of parasympathetic fibers from the greater (superficial) petrosal nerve and sympathetic fibers originating from the deep petrosal nerve, which innervates the lacrimal glands and the mucosa of the upper aerodigestive tract.^3^, ^4^

Anatomically the Vidian nerve canal is located in the sphenoid sinus floor, lateral to the pterygopalatine canal, and extends the pterygopalatine fossa to the foramen lacerum. In small tumors, this anatomy can be appreciated. However, tumors of the Vidian nerve canal tend to be bulky at diagnosis and the anatomy of the region is often distorted.

Of the cases described in the literature, the most common complaint was headache and only one patient had symptoms related to the Vidian nerve function, which was the feeling of dryness in the hard and soft palate. No patient had lacrimal or nasal secretion abnormalities, which correspond to the main functions of the Vidian nerve. Two of the six reported cases were incidental findings and one case was a bilateral Vidian nerve schwannoma.^1^ Our patient had hypoesthesia in the territory of V2 and V3 by compressive effect of the tumor, which was confirmed intraoperatively.

The diagnostic hypothesis of schwannoma can be presumed by the radiological characteristics of the lesion, including contrast enhancement pattern, bone remodeling and Vidian nerve canal enlargement. Surgical resection by endonasal endoscopic surgery was the preferred approach, according to the literature.^2^, ^4^, ^5^ The surgical procedure is considered the treatment of choice for symptomatic lesions and the gold standard for diagnosis. The expectant conduct or radiotherapy may be considered, depending on the size of the lesion and the patient's symptoms.

## Final comments

We report on a rare case that should be considered a differential diagnosis in ventral skull base lesions and, as the other cases described in the literature, the clinical characteristics were not indicative of Vidian nerve dysfunction. Surgical endoscopic treatment through transnasal access allowed complete lesion removal with minimal morbidity.

## Conflicts of interest

The authors declare no conflicts of interest.
